# Late Neurosyphilis and Tertiary Syphilis in Guangdong Province, China: Results from a Cross-sectional Study

**DOI:** 10.1038/srep45339

**Published:** 2017-03-24

**Authors:** Weiming Tang, Shujie Huang, Lei Chen, Ligang Yang, Joseph D. Tucker, Heping Zheng, Bin Yang

**Affiliations:** 1 Dermatology Hospital of Southern Medical University, Guangzhou, China; 2 Guangdong Provincial Dermatology Hospital, Guangzhou, China; 3 Guangdong Center for Skin Diseases and STI Control, Guangzhou, China; 4 University of North Carolina Project-China, Guangzhou, China

## Abstract

Due to challenges in diagnosis and the need for complex laboratory tests, misdiagnosis of neurosyphilis and tertiary syphilis is common in China. We validated the diagnosis and examined the treatment of late neurosyphilis and tertiary syphilis in Guangdong Province, China. A cross-sectional study was conducted to collect data from late neurosyphilis and tertiary syphilis cases reported between 2009 and 2014 in Guangdong, China. Descriptive analysis, bivariate analyses and multiple logistic regressions were performed to determine the structural factors associated with correct diagnosis and standard treatment of late neurosyphilis and tertiary syphilis. Among the 3805 respondents (3805/3936, 96.7%), 1,837 (48.3%) met the misdiagnosed criteria. The misdiagnosis rate decreased over the study period (54.2% in 2009 and 41.8% in 2014). Only 27.1% and 24.9% of the correctly diagnosed late neurosyphilis and tertiary syphilis cases received standard treatment, respectively. Multiple logistic regression revealed that departments of dermatology or STDs [aOR = 3.24, 95% CI: 2.66–3.95], county or township level hospitals [aOR = 2.89, 95% CI: 2.14–3.89], and hospitals situated outside of Pearl River Delta area [aOR = 1.70, 95% CI: 1.46–1.97] had higher likelihood in misdiagnosis of neurosyphilis, compared to the reference groups. Targeted trainings for physicians and expanded syphilis screening services are urgently needed.

Syphilis remains prevalent today, and its incidence is again on the rise^1^. In China, the total number of newly reported syphilis cases was 444,952 (34 per 100,000 persons) in 2013^2^,^3^. The increasing burden of syphilis could potentially increase the pool of people progressing to neurosyphilis and tertiary syphilis. This transition has been observed in China, where the total reported incidence of neurosyphilis and tertiary syphilis also increased, from 0.02 cases per 100,000 persons in 2000 to 0.26 cases per 100,000 persons in 2013^3^. However, the incidence reported in China relies on case reports, which may largely underestimate the overall burden of neurosyphilis.

In addition, due to challenges in diagnosis and the need for complex laboratory tests, misdiagnosis of either neurosyphilis or tertiary syphilis is very common^4^,^5^, particularly in resource limited settings. Neurosyphilis and other tertiary syphilis presents with a wide range of clinical symptoms and are often mistaken for different diseases. For example, one study conducted in 2013 in China reported that six neurosyphilis cases successfully mimicked viral encephalitis^6^. Given these challenges, a large number of neurosyphilis and tertiary cases that are reported to the Chinese Case Report System (CCRS) may be misdiagnosed. For the same reasons, the reported rate of neurosyphilis and tertiary syphilis, which are based on case reports, may be inaccurate.

We validated the diagnosis and treatment of late neurosyphilis and tertiary syphilis in Guangdong Province. In addition, we also aimed to evaluate the factors correlated with misdiagnosis and mistreatment of late neurosyphilis and tertiary syphilis in Guangdong, China.

## Methods

### Study design and sampling methods

A cross-sectional study was conducted to collect data from late neurosyphilis and tertiary syphilis cases reported to the CCRS between January 1, 2009 and December 31, 2014 in Guangdong, China. In China, both late stage neurosyphilis and tertiary syphilis cases identified at any hospital must be reported to the CCRS, and these two diseases were all reported as tertiary syphilis. Thus, in our study, tertiary syphilis included late stage neurosyphilis as well as other subtypes of tertiary syphilis. Information on gender, age, occupation, residential address, and onset date of diagnosis for each case was collected and reported.

In our study, late neurosyphilis was defined as neurosyphilis with no clear evidence of being acquired syphilis within the previous two years, while neurosyphilis was defined as a case with: 1) history of primary, secondary, or latent syphilis history, and 2) clinical manifestations involving central nervous system (CNS), 3) laboratory confirmation (with either a reactive Cerebrospinal Fluid - Venereal Disease Research Laboratory (CSF-VDRL) test or a CSF white blood cells (WBC) count of >20 cells/μL)^7^. To simplify terminology, we will call all late neurosyphilis as neurosyphilis. Tertiary syphilis was defined as a with: 1) history of primary, secondary, or latent syphilis, and 2) clinical manifestations involving the cardiovascular, CNS, Skin and mucous membrane, bone, eye or other organs, and 3) laboratory confirmation with reactive non-treponemal tests, or cerebrospinal fluid abnormalities characterized by higher than normal amounts of white blood cells or protein^8^. In order to be consider to be correctly diagnosed disease, each subtype of neurosyphilis or tertiary syphilis need to have sub-type related clinical manifestations. For example, to be defined as cardiovascular syphilis case, each case need to has syphilitic aortitis.

If the reported late neurosyphilis or tertiary syphilis met the diagnostic criteria of *Guidelines for Diagnosis and Treatment of Sexual Transmitted Diseases* published by National Center for Sexual Transmitted Diseases (STD) Control, China Center for Diseases Control and Prevention (CDC), it was defined as a correct diagnosis^9^. These diagnostic criteria are also similar to the *2015 Sexually Transmitted Diseases Treatment Guidelines* of US CDC^10^. Similarly, standard treatment was defined as late neurosyphilis or tertiary syphilis cases that received the standard treatment suggested by *Guidelines for Diagnosis and Treatment of Sexual Transmitted Diseases*^9^ and *2015 Sexually Transmitted Diseases Treatment Guidelines* of US CDC^10^. If the reported cases did not meet the diagnostic criteria, we further checked the reasons why cases failed to meet the definition.

For the survey, we first downloaded the list of call cases identified and reported during the study period through the CCRS. We then distributed the list of cases to the 21 prefecture-level skin disease and STD control centres, based on the location from which the reports came from. Trained interviewers contacted the hospitals that reported cases and filled out structured questionnaires by checking all of the existing medical records with the help of physicians who diagnosed the cases.

### Measures

A structured questionnaire was used to collect information regarding socio-demographic characteristics, personal syphilis infection history, clinical symptoms, treatment provided, and laboratory testing results of the cases. Socio-demographic information included age (continuous and further categorized to < = 30, 31–40, 41–50, 51–60, 61–70 and >70 years), marital status (married, unmarried, divorced or widowed), occupation (farmer, merchant, retired, home stay, unknown and others), department where diagnosis was made (psychiatry, dermatology or STDs, cardiovascular, orthopedics or eye, others), hospital level (provincial, city, county or township), year of diagnosis (2009–2010, 2011–2012, 2013–2014) and region (Pearl River Delta area, others). We also collected information on the subtype of tertiary syphilis (such as neurosyphilis), damage to the skin or mucosal membranes, osseous syphilis, ocular syphilis and cardiovascular syphilis. We checked all the existing medical records of the reported cases to check whether they were correctly diagnosed. If the cases were correctly diagnosed, we further checked whether they received standard treatment, based on the above mentioned guideline^9^.

### Statistical analysis

EpiData 3.0 was used for double data entry. All analyses were carried out using SAS statistical software version 9.4 (SAS Ins., NC, USA). Descriptive analysis was used to describe the demographic and socio-economic characteristics and the sub-types of correctly reported neurosyphilis and tertiary syphilis. To further evaluate the correlates of misdiagnosis among the reported neurosyphilis and tertiary cases, bivariate analyses were conducted using simple logistic regressions. The bivariate analysis measured the strength of association and the likelihood of being diagnosed correctly in reference to other misdiagnosis cases. In addition, multiple logistic regressions were conducted to determine the factors associated with misdiagnosis and standard treatment. The adjusted odds ratios (aORs) and corresponding 95% confidence intervals (CIs) were also calculated. In these models, we adjusted for age (continuous), marital status (married/never married/divorced or widowed) and occupation (farmer/ merchant/ retired/ home stay/ unknown/others). Correctly reported status (correct diagnosis or misdiagnosis, dichotomous) was treated as the dependent variable, while other variables were treated as independent variables. We also evaluated the factors associated with standard treatment among correctly diagnosed and reported late neurosyphilis and tertiary syphilis cases, separately.

### Ethics statement

The study process and contents were approved by the Ethics Committee of the Guangdong Provincial Center for Skin Disease and STDs Control (20150311). Oral informed consent was obtained from the participants through telephone call. The questionnaires were separately kept in locked cupboards at the study sites, and unauthorized access was not possible. All methods in this study were performed in accordance with the approved process.

## Results

During the study period, a total of 297,782 syphilis cases were reported to the CCRS between 2009 and 2014, while 3,936 (1.3%) of them were reported as tertiary syphilis cases (Appendix A). Between April and October of 2015, we successfully followed up with and collected further information from 3,805 of these cases, with a response rate of 96.7%.

Of the 3,805 cases, the mean age of the patients was 52.5 ± 16.9 years old. One third (32.4%) of the patients were greater than 60 years old. In addition, the majority of the participants were married (90.3%), about a quarter of them were farmers (25.8%), and about one fifth of the cases reported their primary job as stay at home (19.9%) (Table 1).

More than two fifths (42.6%) of the participants were diagnosed and reported by the psychiatry department of different hospitals, and about one sixth (16.3%) of the participants were diagnosed and reported by dermatology or STDs departments.

A majority of the cases were reported by the city level hospitals (69.9%), while county level or township level hospitals reported about one quarter of the cases (23.4%). The yearly number of reported cases was similar during the study period (2009–2014). In addition, about three-tenths (28.9%) of the reported cases were reported by hospitals situated in the Pearl River Delta area, which included nine of the 21 prefecture-level cities in Guangdong Province (Table 1).

### Misdiagnosis

Based on the existing medical records and the diagnostic STD criteria^9^, a total of 1,837 cases met the misdiagnosed criteria (48.3%, with no clinical manifestations of tertiary syphilis). We further checked the medical results of the 1,837 misclassified cases, and found that 28 (1.5%) cases can be considered as primary syphilis (Trust and TPPA positive, and with symptoms of primary syphilis), 23 (1.3%) cases as secondary syphilis (Trust and TPPA positive, and with secondary syphilis symptoms), 1,106 (60.2%) cases can be considered as latent syphilis (Trust and TPPA positive, but no syphilis related clinical manifestations, or Trust positive but TPPA negative and without syphilis related clinical manifestations), and 680 (37.0%) cases can be considered as syphilis negative (Both Trust and TPPA tests are negative) (Appendix B).

**Figure 1:**
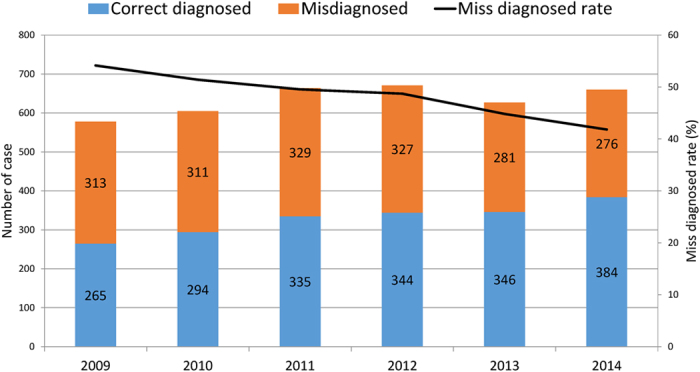
Trend of misdiagnosis of tertiary syphilis in Guangdong, China, 2009–2014.

The misdiagnosis rate consistently decreased over the study period, from 54.2% (313/578) in 2009 to 41.8% (276/660) in 2014 (*p* for trend < 0.001) (Fig. 1).

### Neurosyphilis

Among the correctly diagnosed cases, 1,615 (82.1%) met the diagnostic criteria of neurosyphilis and were consider to be late neurosyphilis cases. The most common subtype of neurosyphilis cases were paresis (68.4%), followed by meningovascular syphilis (11.5%) and syphilitic meningitis (11.1%) (Table 2). The neurosyphilis cases only accounted for 0.54% (1,615/297,782) of the overall reported syphilis cases.

After removing the misdiagnosed cases, the incidence rate of neurosyphilis is still increasing [increased from 0.21 cases per 100,000 persons (202/95,432,603) in 2009 to 0.31 cases per 100,000 persons (327/106,443,914) in 2014]. Overall, 70.5% of the neurosyphilis cases received treatment after the diagnosis. However, only 27.1% of them received the standard treatment.

### Tertiary syphilis

Of the 1,968 correctly diagnosed tertiary syphilis, the most common subtype was neurosyphilis (82.1%), followed by cardiovascular syphilis (7.4%, with syphilitic aortitis), involvement of the skin mucosal membranes (6.1%) and osseous syphilis (2.6%). Correctly diagnosed tertiary syphilis cases only accounted for 0.66% (1,968/297,782) of the overall reported syphilis cases during the study period.

**Figure 2:**
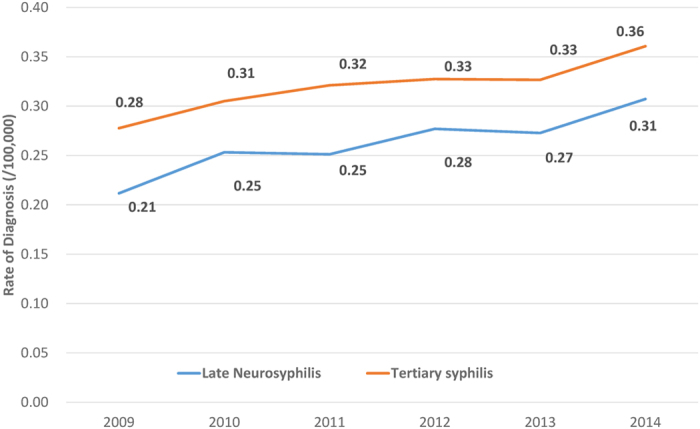
Trend of population level diagnosis rate of neurosyphilis and all tertiary syphilis in Guangdong, Chia, 2009–2014.

The overall rate of tertiary syphilis increased from 0.28 cases per 100,000 persons (265/95,432,603) in 2009 to 0.36 cases per 100,000 persons (384295/106,443,914) in 2014 (Fig. 2). However, only 65.7% of the tertiary cases received treatment after the diagnosis, and only 27.3% of them received the standard treatment.

### Correlates of misdiagnosis

Results from binary logistic regression indicated that compared to psychiatry departments, departments of dermatology/STDs, cardiovascular/orthopedics/eyes, and other departments had a higher likelihood of misdiagnosis of neurosyphilis or other tertiary syphilis, with crude ORs (cORs) of 3.13 [95% confidence interval (CI): 2.59–3.80], 5.40 (95% CI: 4.10–7.11), and 5.24 (95% CI 4.47–6.14), respectively. In addition, compared to provincial level hospitals, county or township level hospitals had a higher likelihood of misdiagnosis cases, with cOR of 2.92 (95% CI: 2.19–3.89). Our results also indicated that compared to 2013–2014, 2009–2010 and 2011–2012 had significantly higher levels of misdiagnosis, with cORs of 1.46 (95% CI: 1.25–1.72) and 1.27 (95% CI: 1.09–1.48), respectively. Compared to hospitals in the Pearl River Delta area, hospitals in other regions had a higher likelihood in case misdiagnosis, with cOR of 1.77 (95% CI: 1.54–2.04). Similar results were found in the multivariable logistic regression models, after we adjusted for participant’s age, marital status and occupation.

### Correlates of standard treatment among neurosyphilis cases

Results from multivariable logistic regression indicated that compared to psychiatry departments, late neurosyphilis cases identified at dermatology or STDs departments, and other departments were more likely to receive standard treatment, with adjusted ORs (aORs) of 2.23 (95% CI: 1.59–3.13) and 2.01 (95% CI: 1.52–2.66), respectively. In addition, compared to provincial level hospitals, neurosyphilis cases identified at city level hospitals were more likely to receive standard treatment, with aOR of 3.68 (95% CI: 1.74–7.80). Neurosyphilis cases identified between 2011–2012 were less likely to receive standard treatment, compared to patients identified between 2013–2014 (aOR of 0.71, 95% CI: 0.54–0.92). Neurosyphilis cases identified outside of the Pearl River Delta area were also less likely to receive standard treatment, with aOR of 0.76 (95% CI: 0.57–1.02) (Table 3).

### Correlates of standard treatment among tertiary syphilis cases

Similar to the neurosyphilis cases, tertiary syphilis cases identified at dermatology or STDs departments, and other departments were more likely to receive standard treatment, with aORs of 2.62 (95% CI: 1.98–3.46) and 1.64 (95% CI 1.27–2.10), respectively. In addition, compared to cases identified at provincial level hospitals, tertiary syphilis cases identified at city level hospitals were more likely to receive standard treatment, with aOR of 1.65 (95% CI 1.08–2.50). Similarly, tertiary syphilis identified between 2011–2012 were less likely to receive standard treatment, with aOR of 0.78 (94% CI 0.56–0.90). Compared to tertiary syphilis identified in Pearl River Delta area, cases identified outside of the Pearl River Delta area were less likely to receive standard treatment, with aOR of 0.77 (95% CI: 0.60–0.99) (Table 4).

## Discussion

Our study evaluated the medical records of 3,885 neurosyphilis and other tertiary syphilis cases across all cities in Guangdong, China between 2009 and 2014. Our study demonstrated that there is a heavy burden of neurosyphilis and overall tertiary syphilis in Guangdong and it is increasing. In addition, misdiagnosis of neurosyphilis and all tertiary syphilis, as well as lack of standard treatment in China, are problematic. Our study extends upon previous literature by evaluating medical records of all reported neurosyphilis and other tertiary syphilis cases documented in the CCRS from Guangdong province between 2009 and 2014. It evaluates the misdiagnosis of the reported cases and assesses the treatment regimen among correctly diagnosed late neurosyphilis and among all tertiary syphilis cases in China.

Our study showed that a total of 1,968 tertiary syphilis cases were correctly diagnosed during the study period. Overall, the rate of neurosyphilis diagnosis in Guangdong (0.31 cases per 100,000 persons) was lower than the rate reported in British Columbia in Canada (0.8 cases per 100,000 persons in 2012). However, because our study was merely based on case reports, a large proportion of the neurosyphilis and tertiary syphilis cases may not have been included. As a result, the overall burden of neurosyphilis and tertiary syphilis in China could be much higher compared to Canada and other Western countries. There is some evidence that there is a high overall burden of syphilis in China^3^,^11^.

Increasing reported neurosyphilis and tertiary syphilis rates were observed in Guangdong during the study period. Several potential reasons may have contributed to the increasing burden of neurosyphilis and tertiary syphilis. First, the increasing epidemic of neurosyphilis and tertiary syphilis in Guangdong and across China stems from an increasing pool of patients infected with syphilis^12^. Second, lack of screening and poor service delivery have contributed to the epidemic. For example, one survey from 2013 revealed that only about 30% men who have sex with men (MSM) reported ever having tested for syphilis, despite a syphilis prevalence of 9%^13^. Limited screening and other related services may miss a large number of syphilis patients that need to be diagnosed and treated, resulting in a larger proportion of preventable early syphilis cases progressing to neurosyphilis or other types of tertiary syphilis. Third, stigma and social discrimination may play important roles in the epidemic. According to a recent UNAIDS report, stigma and social discrimination were found to be two of the greatest barriers to successful implementation of STDs prevention and control globally^14^. In China, these factors are more pronounced due to cultural and historical unacceptability of STDs, particularly syphilis. The stigma and social discrimination deeply influences individuals’ health-seeking behaviors^15^, and leads to delays in seeking and obtaining syphilis diagnosis and treatment^16^. As a result, a greater proportion of syphilis cases progress to neurosyphilis and tertiary syphilis. Many studies and reports recommend that physicians should screen neurosyphilis in patients with clinical signs and symptoms of central nervous system involvement, HIV infection, serum titer > = 1:32 or with documented treatment failure of syphilis^10^,^17^,^18^. It has been advised that China should adapt the same recommendation and should initiate screening as early as possible.

Our study also indicated that around half of reported cases were misdiagnosed as neurosyphilis or other tertiary syphilis cases, which is significant. One important reason for this phenomenon is that the diagnosis of neurosyphilis and tertiary syphilis is challenging. Previous studies indicated that syphilis can mimic several other diseases, which makes differentiating neurosyphilis and tertiary syphilis from other diseases with similar symptoms^12^,^19^ difficult. In addition, the diagnosis of neurosyphilis and tertiary syphilis strongly relies on clinical features, stage characteristics, epidemiological history of the patients, and laboratory testing results^20^,^21^. It is hard for institutes in resource limited setting with inadequately staffed laboratories to provide an accurate diagnosis of neurosyphilis and tertiary syphilis^21^. Our study results indicated that hospitals in the regions outside of the Pearl River Delta area and county/township level hospitals had significantly higher misdiagnosis rates.

Even when correctly diagnosed, only two-thirds of neurosyphilis and tertiary syphilis cases received treatment, and only around one-quarter of cases received standard treatment. The low rates of both treatment and standard treatment for neurosyphilis and tertiary syphilis is a significant problem for patients who continue to present with symptoms and suffer from the illness^22^,^23^. Our study also indicated that the psychiatry departments in different hospitals had a much lower likelihood in providing standard treatment for patients, despite having a higher likelihood in correctly diagnosing neurosyphilis and tertiary syphilis. These findings indicate that increased training should be provided to physicians in those departments. In addition, hospitals outside of the Pearl River Delta area should require additional training for physicians to improve diagnosis and treatment of late neurosyphilis and tertiary syphilis.

Our study also suffers from several limitations. First, our study may have selection bias induced by non-response, although our study successfully reached 96.7% of all the reported cases. Second, we only checked available medical reports. We did not collect further information from patients. Third, the diagnosis of neurosyphilis and tertiary syphilis is challenging, and our study may suffer from outcome misclassification. For example, the manifestations of tertiary syphilis can be very confusing, and this confusing may lead to misdiagnosis of the tertiary syphilis cases. Fourth, our study only evaluated the problem of misdiagnosis. The impact of missed detection and misreporting is still unknown, and the real epidemic may be uncovered by improving missed detection. Fifth, even the physicians did distinguish co-existing cardiac disease or CNS disease that had a different etiology from cardiac or CNS disease at the diagnosis stage, these data were not collected. This prevented us to further distinguish these diseases, and may lead to outcome misclassification. Last but not least, the CCRS only reported late neurosyphilis (reported as tertiary syphilis), while early neurosyphilis was excluded from the current reported study. Therefore, we could not assess early neurosyphilis cases. However, the proportion of early neurosyphilis cases is believed to be very low.

In conclusion, our study found high levels of misdiagnosis and inadequate treatment of neurosyphilis and tertiary syphilis cases in Guangdong, China. Targeted trainings focused on improving the capacity of physicians in related hospital departments are urgently needed. In addition, we should further expand syphilis screening services in order to identify and treat cases in earlier stages. Further epidemiological studies are needed to better understand misdiagnosis rates, disease burden, and determinants of neurosyphilis and overall tertiary syphilis.

## Additional Information

**How to cite this article:** Tang, W. *et al*. Late Neurosyphilis and Tertiary Syphilis in Guangdong Province, China: Results from a Cross-sectional Study. *Sci. Rep.* **7**, 45339; doi: 10.1038/srep45339 (2017).

**Publisher's note:** Springer Nature remains neutral with regard to jurisdictional claims in published maps and institutional affiliations.

## Supplementary Material

Supplementary Appendix

## Figures and Tables

**Table 1:** Demographic and Hospital Information for reported Tertiary cases in Guangdong, China, 2009–2014 (N = 3805).

		Correct diagnosed (n = 1968)		Misdiagnosed (n = 1837)		Overall (N = 3805)	
Variables		Number	Percent (95% CI)	Number	Percent (95% CI)	Number	Percent
Age	30 or less	91	4.6 (3.7,5.6)	248	13.5 (11.9,15.1)	339	8.9
	31–40	216	10.8 (9.6,12.4)	270	14.7 (13.1,16.3)	486	12.8
	41–50	519	26.4 (24.4,28.3)	298	16.2 (14.5,17.9)	817	21.5
	51–60	579	29.4 (27.4,31.4)	349	19.0 (17.2,20.8)	928	24.4
	61–70	345	17.5 (15.8,19.2)	310	16.9 (15.2,18.6)	655	17.2
	Above 70	218	11.1 (9.7,12.5)	362	19.7 (17.9,21.5)	580	15.2
Marital Status	Married	1784	90.6 (89.4,91.9)	1653	90.0 (88.6,91.4)	3437	90.3
	Unmarried	109	5.5 (4.5,6.6)	129	7.0 (5.8,8.2)	238	6.2
	Divorced or Widowed	75	3.8 (3.0,4.6)	55	3.0 (2.2,3.8)	130	3.4
Occupation	Farmer	474	24.1 (22.2,26.0)	507	27.6 (25.6,29.6)	981	25.8
	Merchant	138	7.0 (5.9,8.1)	121	6.6 (5.4,7.7)	259	6.8
	Retired	292	14.8 (13.3,16.4)	225	12.2 (10.74,13.7)	517	13.6
	Stay at home	422	21.4 (19.6,23.2)	335	18.2 (16.5,20.0)	757	19.9
	Unknown	275	14.0 (12.4,15.5)	339	18.4 (16.7,20.2)	614	16.1
	Others	367	18.6 (16.9,20.4)	310	16.9 (15.2,18.6)	677	17.8
Department of diagnosis	Psychiatry	1171	59.5 (57.3,61.7)	451	24.6 (22.6,26.5)	1622	42.6
	Dermatology or STDs	281	14.3 (12.7,15.8)	339	18.4 (16.7,20.2)	620	16.3
	Cardiovascular, orthopedics or eye	89	4.5 (3.6,5.4)	185	10.1 (8.7,11.4)	274	7.2
	Others	427	21.7 (19.9,23.5)	862	46.944.6,49.2)	1289	33.9
Hospital level	Provincial	147	7.6 (6.4,8.7)	104	5.8 (4.8,6.9)	251	6.7
	City	1513	77.8 (76.0,79.7)	1086	61.1 (58.9,63.4)	2599	69.9
	County or township	284	14.6 (13.0,16.2)	586	33.0 (30.8,35.2)	870	23.4
Year	2009–2010	559	28.4 (26.4, 30.4)	624	34.0 (31.8,36.1)	1183	31.1
	2011–2012	679	34.5 (32.4, 36.6)	656	35.7 (33.5,37.9)	1335	35.1
	2013–2014	730	37.1 (35.0,39.2)	557	30.3 (28.2, 32.4)	1287	33.8
Region	Pearl River Delta area	458	23.3 (21.4, 25.1)	642	34.9 (32.8, 37.1)	1100	28.9
	Others	1510	76.7 (74.8, 78.6)	1195	65.1 (62.9, 67.2)	2705	71.1

**Table 2:** Sub-types of correctly diagnosed tertiary syphilis in Guangdong, China, 2009–2014 (n = 1968).

Disease type	Number of cases	Percent (%)
Neurosyphilis (Overall)	1615	82.1
Syphilitic meningitis*	179	11.1
Syphilitic pachymeningitis*	53	3.3
Meningovascular syphilis*	186	11.5
Paresis*	1105	68.4
Tabes dorsalis*^,#^	115	7.1
Optic atrophy*	73	4.5
Damage of skin mucous membrane	120	6.1
Osseous syphilis	51	2.6
Ocular syphilis	41	2.1
Cardiovascular syphilis^*,#*^	145	7.4
Other	40	2

Note: *Subtypes of neurosyphilis, and the denominator is 1615.

^#^For the cases of cardiovascular syphilis and tabes, the patients need to have the clinical manifestations involving the cardiovascular and CNS.

**Table 3:** Correlates of Misdiagnosis among reported tertiary cases in Guangdong in 2009–2014, China (N = 3,805).

		Crude Model			Adjusted Model*		
Variables		Crude OR	95% CLs		Adjusted OR	95% CLs	
Department of diagnosis	*Psychiatry*	*Ref*			*Ref*		
	*Dermatology or STDs*	3.13	2.59	3.80	3.24	2.66	3.95
	*Cardiovascular, orthopedics or eye*	5.40	4.10	7.11	5.51	4.16	7.28
	*Others*	5.24	4.47	6.14	5.36	4.56	6.29
Hospital level	*Provincial*	—			—		
	*City*	1.02	0.78	1.32	1.00	0.77	1.31
	*County or township*	2.92	2.19	3.89	2.89	2.14	3.89
Year	*2009–2010*	1.46	1.25	1.72	1.44	1.22	1.69
	*2011–2012*	1.27	1.09	1.48	1.28	1.09	1.49
	*2013–2014*	—			—		
Region	*Other Regions*	1.77	1.54	2.04	1.70	1.46	1.97
	*Pearl River Delta*	—			—		

Note: *adjusted for age (continuous), marital status (married/never married/divorced or widowed) and occupation (Farmer/ Merchant/ Retired/ Home stay/ Unknown/Others).

**Table 4:** Correlates of standard treatment among correctly diagnosed neurosyphilis and tertiary syphilis cases in Guangdong in 2009–2014, China (n = 1968).

		Late Neurosyphilis						All tertiary syphilis					
		Crude Model			Adjusted Model*			Crude Model			Adjusted Model*		
		cOR	95% CLs		aOR	95% CLs		cOR	95% CLs		aOR	95% CLs	
Department of diagnosis	*Psychiatry*	Ref			Ref			Ref			Ref		
	*Dermatology or STDs*	2.33	1.68	3.24	2.23	1.59	3.13	2.81	2.14	3.69	2.62	1.98	3.46
	*Cardiovascular, orthopedics or eye*	1.23	0.48	3.16	1.32	0.51	3.43	1.10	0.66	1.83	1.28	0.76	2.15
	*Others*	2.13	1.62	2.81	2.01	1.52	2.66	1.66	1.30	2.13	1.64	1.27	2.10
Hospital level	*Provincial*	—			—			—			—		
	*City*	3.49	1.66	7.35	3.68	1.74	7.80	1.49	0.99	2.24	1.65	1.08	2.50
	*County or township*	1.46	0.64	3.35	1.62	0.70	3.77	0.94	0.58	1.53	1.10	0.67	1.82
Year	*2009–2010*	0.87	0.67	1.14	0.86	0.66	1.14	0.88	0.69	1.12	0.84	0.65	1.07
	*2011–2012*	0.69	0.53	0.90	0.71	0.54	0.92	0.71	0.56	0.90	0.78	0.56	0.90
	*2013–2014*	—			—			—			—		
Region	*Others*	0.66	0.50	0.87	0.76	0.57	1.02	0.74	0.58	0.94	0.77	0.60	0.99
	*Pearl River Delta*	—						—					

Note: *adjusted for age (continuous), marital status (married/never married/divorced or widowed) and occupation (Farmer/ Merchant/ Retired/ Home stay/ Unknown/Others).
