# Supplementary material for: Late Neurosyphilis and Tertiary Syphilis in Guangdong Province, China: Results from a Cross-sectional Study
Source: Sci Rep. 2017 Mar 24;7:45339. doi: 10.1038/srep45339 (PMC5364404; doi:10.1038/srep45339)
Supplement: Supplementary Appendix [file srep45339-s1.doc]

**Late Neurosyphilis and Tertiary Syphilis in Guangdong Province, China: Results from a Cross-sectional Study**

Weiming Tang^1,2,3,4,*^, Shujie Huang^1,2,3,*^, Lei Chen^1,2,3^, Ligang Yang^1,2,3^, Joseph D. Tucker^4^, Heping Zheng^1,2,3^, Bin Yang^1,2,3,#^

**Appendix**

**Appendix A: Syphilis Epidemic in Guangdong, China, 2009-2014 (N=297,782)**

| Year | Reported Incidence | | No. of reported cases | | | Cases meeting diagnostic criteria |
| --- | --- | --- | --- | --- | --- | --- |
|  | All syphilis | Tertiary syphilis | All syphilis | Tertiary syphilis | Proportion of tertiary syphilis |  |
| 2009 | 40.98 | 0.54 | 39,112 | 514 | 1.31 | 265 |
| 2010 | 47.10 | 0.69 | 45,399 | 668 | 1.47 | 294 |
| 2011 | 48.94 | 0.67 | 51,051 | 698 | 1.37 | 335 |
| 2012 | 50.49 | 0.68 | 53,043 | 716 | 1.35 | 344 |
| 2013 | 50.26 | 0.63 | 53,241 | 671 | 1.26 | 346 |
| 2014 | 52.55 | 0.63 | 55,936 | 669 | 1.20 | 384 |
| Overall | 48.53 | 0.64 | 297,782 | 3936 | 1.32 | 1968 |

**Appendix B: Reasons for the cases failing to meet the diagnostic criteria of tertiary syphilis in Guangdong, China, 2009-2014 (n=1837)**

| Reasons | Number | Proportion (%) |
| --- | --- | --- |
| Had symptoms related to primary syphilis and met the diagnostic criteria of primary syphilis | 28 | 1.5 |
| Had symptoms related to secondary syphilis and met the diagnostic criteria of primary syphilis | 23 | 1.3 |
| Both TPPA and Trust test results were positive, but no syphilis related symptoms | 913 | 49.7 |
| Only Trust test result was positive, and no syphilis related symptoms | 193 | 10.5 |
| Both TPPA and Trust test results were negative | 680 | 37.0 |
| Total | 1837 | 100.0 |
